# Supplementary material for: New Composite Contrast Agents Based on Ln and Graphene Matrix for Multi-Energy Computed Tomography
Source: Nanomaterials (Basel). 2022 Nov 22;12(23):4110. doi: 10.3390/nano12234110 (PMC9737213; doi:10.3390/nano12234110)
Supplement: Supplementary file 1 [file nanomaterials-12-04110-s001.zip › nanomaterials-1995095-figures1.pdf]

Supplementary Materials

New Composite Contrast Agents Based on Ln and Graphene Matrix for Multi-Energy Computed Tomography

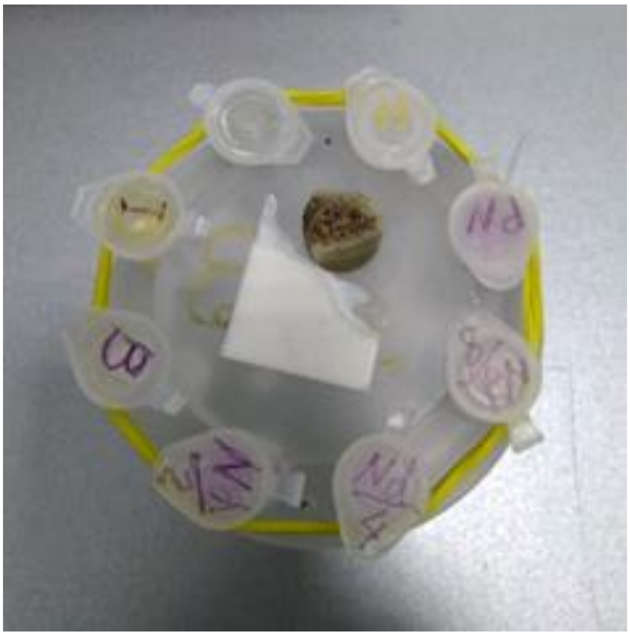

Figure S1. Polystyrene phantom with samples in the Eppendorf tubes and bone. The samples assessed in each specific experiment are specified in subsequent figures.
